# Strategy advancements in placental pharmacokinetics: from *in vitro* experiments to *in silico* prediction

**DOI:** 10.3389/fphar.2025.1694886

**Published:** 2025-10-27

**Authors:** Zhimin Li, Yue Wu, Siyu Zeng, Fei Wang, Jiao Zhang, Shiran Li, Yong Yang, Yujie Yang

**Affiliations:** ^1^ Department of Pharmacy, Personalized Drug Research and Therapy Key Laboratory of Sichuan Province, Sichuan Provincial People’s Hospital, School of Medicine, University of Electronic Science and Technology of China, Chengdu, China; ^2^ Department of Pharmacy, Chongqing Red Cross Hospital (Jiangbei District People’s Hospital), Chongqing, China; ^3^ Department of Pharmacy, Affiliated Hospital of Southwest Jiaotong University, The Third People’s Hospital of Chengdu, Chengdu, China

**Keywords:** pharmacokinetics, placental drug transfer, *in vitro* model, *ex vivo* placental perfusion, *in silico* simulation

## Abstract

**Background:**

The placental barrier is a critical interface that regulates drug transport between maternal and fetal circulation and is an important component in assessing fetal drug-exposure risk. Since pregnant women are often excluded from clinical trials, pharmacokinetic (PK) analysis data on placental drug transport remain limited. Currently, *in vitro* experiments and *in silico* simulation strategies are the primary and effective means for understanding drug transport across the placenta.

**Method:**

Various *in vitro* experimental methods, including cell monolayer models, *ex vivo* placental perfusion, and organ-on-a-chip platforms, along with model-based computational simulations, were systematically reviewed. The advantages, limitations, and potential future applications of these methods were evaluated.

**Result:**

A total of seven studies using cell models, 28 employing *ex vivo* perfusion, six utilizing placenta-on-a-chip technology, and 39 focusing on *in silico* simulations, were identified, involving 8, 34, 5, and 42 drugs, respectively. Antiviral agents, antibiotics, and opioids were the most frequently investigated drug types. Overall, *in silico* simulations informed by *in vitro* data as baseline parameters and constraints demonstrated higher predictive accuracy. Integrating multi-model data was shown to be a reliable strategy for improving the precision of placental PK studies.

**Conclusion:**

This review highlights the current strategies in placental PK research and supports safer drug use during pregnancy. Multi-model data integration is essential for developing reliable and quantitative fetal drug-exposure assessment frameworks, thus addressing data gaps caused by the exclusion of pregnant women from clinical trials.

## Highlights

- This review systematically examined state-of-the-art *in vitro* modeling methods, including cell monolayer models, *ex vivo* placental perfusion experiments, organ-on-a-chip platforms, and model-guided computer simulations, and it critically appraised their respective strengths and limitations in quantifying transplacental drug transfer.
- Physiologically based pharmacokinetic (PBPK) models simulate the absorption, distribution, metabolism, and excretion (ADME) of drugs and are a powerful tool for predicting fetal drug exposure during pregnancy.
- The implementation of an integrated multi-model approach significantly enhances the reliability of maternal–fetal pharmacokinetic (PK) assessments, providing robust evidence to support guideline updates and clinical decision-making during pregnancy.

## 1 Introduction

Many pregnant women require one or more medications for health problems (Be et al., 2025; Lupattelli et al., 2014). Drug use during pregnancy is on the rise (Subramanian et al., 2023; Phillips et al., 2024), and recent studies have shown an increase in the use of at least one prescribed medication from 56.9% in 1998 to 63.3% in 2018 and an increase in the concurrent use of multiple medicines from 24.8% to 35.2% in the Danish population (Thunbo et al., 2024). Although drugs have therapeutic significance in clinical practice, their safety in mothers and fetuses remains unclear. Maternal drug use may affect the fetus, leading to developmental abnormalities (Foster et al., 2022; De Felice and Kane, 2021) or long-term health issues (Abolhassani et al., 2023; Owen et al., 2021). Therefore, fetal drug exposure and safety assessments have gradually become important topics in clinical pharmacology.

The effects of maternal drug use on the fetus are complex and sensitive and require a multifaceted assessment. The syncytiotrophoblast (STB) and its two polarized plasma membranes, the maternal-facing microvillous membrane (MVM) and fetal-facing basal membrane (BM), represent the primary barrier in the human placenta, controlling transplacental transfer of small solutes. Key factors, including the physicochemical properties of the drug (MW, logP, pKa, protein binding, transporters, and metabolism) and placental function at different time points, affect the ability of the drug to cross the placental barrier during pregnancy. Due to the complexity of the placental structure, the assessment of transplacental drug transfer is a long-standing and difficult issue. Understanding and quantifying drug transport through the placenta can inform the assessment of fetal drug exposure. However, pregnant women are often excluded from clinical and post-marketing studies. This has led to a lack of PK data regarding drug use during pregnancy, particularly data related to placental drug transfer (Pariente et al., 2016). Therefore, considering the mismatch between the available evidence from existing studies and real-world medication needs during pregnancy (Haas et al., 2018), PK research on placental drug transfer remains a challenging and long-term endeavor.

**FIGURE 1:**
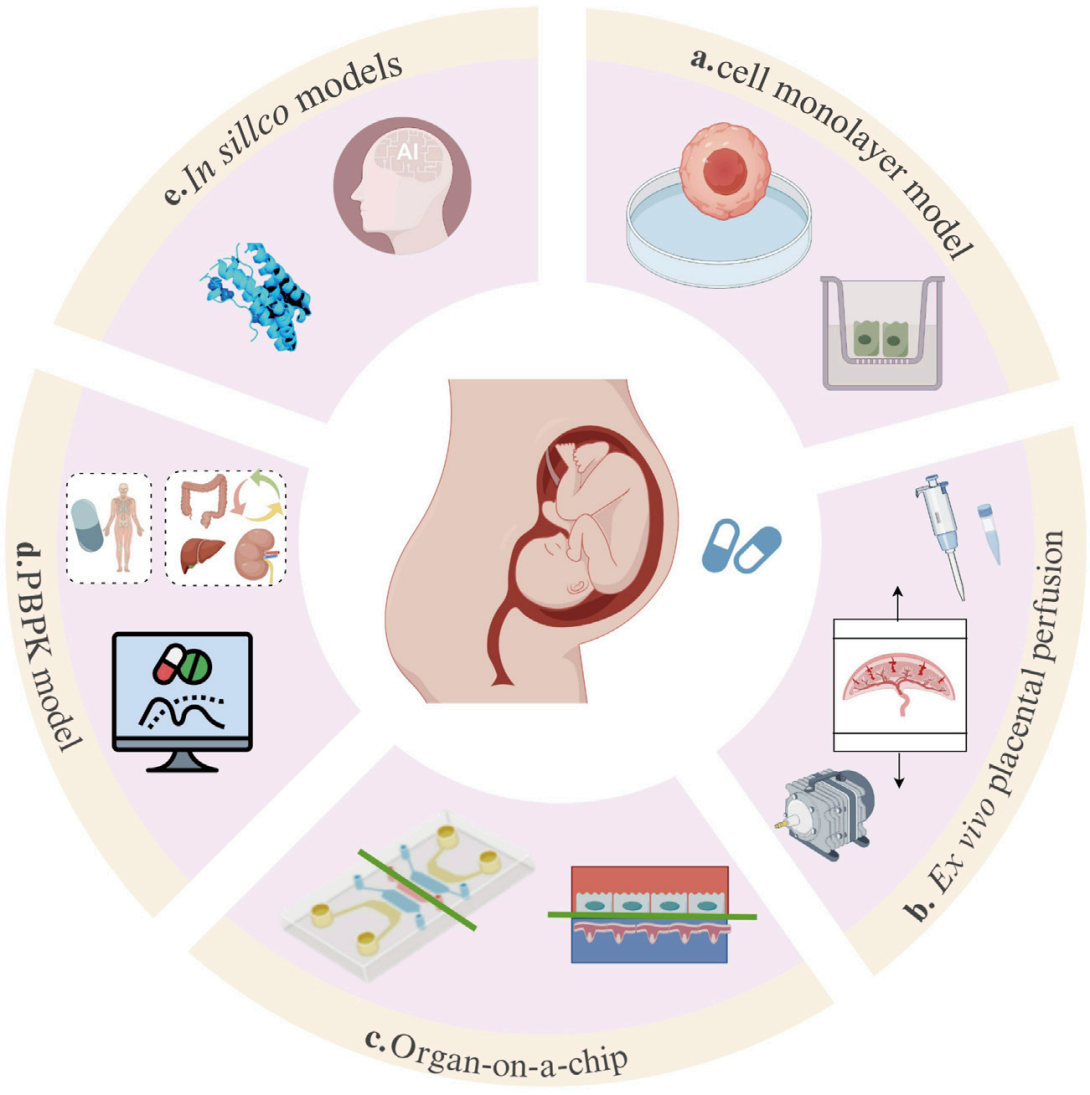
Overview of methods for placental drug-transport studies. The main approaches of placental drug-transport studies include the BeWo/Caco-2 cell model **(a)**, the *ex vivo* placental perfusion experiment **(b)**, the organ-on-a-chip model **(c)**, the PBPK model **(d)**, and *in silico* models **(e)**.

In recent years, with the advancement of computer and bioengineering technologies, methods for placental PK studies have developed considerably. In addition to the previously established *in vitro* methods (e.g., cell monolayer model, Figure 1a; *ex vivo* placental perfusion experiment; Figure 1b), several new attempts have been made (for example, organ-on-a-chip Figure 1c; PBPK model, Figure 1d; *in silico* models, Figure 1e). Multiple approaches have been attempted, including the combination of PBPK models with *in vitro* and *ex vivo* data. However, systematic evaluation and synergistic integration of these methods have not yet been fully explored. This review systematically discusses the evolution, application scenarios, and limitations of various placental drug transfer methods in the context of drug studies. We have also attempted to explore the future directions of these research methods. This study ultimately aims to contribute to the methodological advancement of placental pharmacokinetics and risk assessment for medication use during pregnancy.

## 2 Literature review procedure

A systematic literature search was performed in PubMed (https://pubmed.ncbi.nlm.nih.gov/) from inception to February 2025 using the keywords “placental drug transfer,” “placental pharmacokinetics,” and “foetal drug exposure.” Two team members independently screened the titles and abstracts, and any controversial finding was resolved through discussion with a third researcher. Studies were included if they investigated drug transfer across the human placenta using cell monolayer models, *ex vivo* placental perfusion, organ-on-a-chip platforms, or *in silico* simulations and reported relevant methodological outcomes. Studies not related to the human placenta, not addressing drug transfer across the placenta, lacking original data, or with insufficient methodological details were excluded. Figure 1 summarizes the *in vitro* models applied in transplacental drug transfer research. Detailed information on the progress, advantages, and limitations of each method was reviewed and summarized to highlight the future research directions for *in vitro* human placental models.

## 3 Cell experimental models for placental drug-transfer

Current placental transport models include primary trophoblasts, trophoblast cell lines (BeWo b30, JEG-3, JAR, and HTR-8/SVneo), stem cell-derived systems, and intestinal Caco-2 (cancer coli-2) cells, with BeWo b30 and primary trophoblasts remaining the gold standards according to the FDA/ICH guidelines. We mainly focus on placental barrier studies, with studies on BeWo cells focusing on the mechanism of drug transport across the placenta and the role of transport proteins, while Caco-2 cells are mainly used to assess drug permeability across the placental barrier, and therefore, the two cellular studies will be described in detail below.

### 3.1 Trophoblast cell lines

The BeWo cell line serves as a valuable *in vitro* model for placental transport studies. Derived from human choriocarcinoma, these cells form functional syncytialized monolayers when properly differentiated upon cAMP induction (Drwal et al., 2018), exhibiting key characteristics of the placental barrier (Moore et al., 2021). Syncytialized BeWo cells were employed as an *in vitro* model of the placental barrier to investigate drug-transport kinetics, following verification of barrier integrity through transepithelial electrical resistance (TEER) measurements (≥80 Ω · cm^2^).

The transporters expressed in the STB, including the ABC family members (P-gp, BCRP, and MRP) and SLC family members (OCT and OAT), bidirectionally regulate placental substance exchange through efflux (e.g., P-gp/BCRP/MRP-mediated excretion into maternal circulation) and uptake (e.g., OCT/OAT-mediated substance intake) mechanisms (Bai et al., 2016). BeWo b30 cells, a subclone of the BeWo cell line, expresses important placental transporters (Han et al., 2018), particularly BCRP and P-gp (Ceckova et al., 2006), and it demonstrates compound permeability patterns that correlate well with *ex vivo* results (Correia Carreira et al., 2011; Li et al., 2013; Poulsen et al., 2009). Researchers have successfully utilized this model to initially elucidate the transplacental transport of clinically relevant compounds spanning antivirals, opioids, and fluoroquinolones (Table 1).

**TABLE 1:** Details of placental drug-transfer study by the cell experimental models.

Cell line	Origin	Drug	Type	Key findings	Ref.
Caco-2 cell	Colon cancer cells	Acetaminophen	Quantitative	CLp: 4354	Mian et al. (2020)
	Colon cancer cells	Nifedipine	Quantitative	CLp: 3779	Werdan Romão et al. (2024)
	Colon cancer cells	Vancomycin	Quantitative	CLp: 27	Yan et al. (2024)
BeWo cell	Choriocarcinoma cells	Opioids	Quantitative	P (heroin): 0.21; P (oxycodone): 2.46	Mortensen et al. (2019)
	Choriocarcinoma cells	Abacavir	Descriptive	TTPTs: ENT1	Cerveny et al. (2018)
	Choriocarcinoma cells	Zidovudine Emtricitabine	Descriptive	TPTs: without ENTs	Karbanova et al. (2017)
	Choriocarcinoma cells	Fluoroquinolone	Descriptive	TPTs (LVFX): BCRP、OAT、 MCT	Po et al. (2018)

Abbreviations: CLp (mL/min), transplacental clearance; P (×10^−5^ cm/s): the permeability across the cells; TPTs, placental transporter proteins; ENT, equilibrative nucleoside transporter; LVFX, levofloxacin; BCRP, breast cancer resistance protein; OAT, organic anion transporter; MCT, monocarboxylate transporter.

However, this model has notable limitations. First, a comparative transcriptomic study of human placental *in vitro* models revealed substantial numbers of differentially expressed genes across all *in vitro* placental models. To date, no placental cell line has been able to accurately mimic human placental tissue (Lapehn et al., 2024). Although steroidogenic enzymes such as 3β-hydroxysteroid dehydrogenase type 1 (3β-HSD1) and 11β-hydroxysteroid dehydrogenase type 2 (11β-HSD2) are highly expressed and functionally active in the *in vivo* placenta, their expression levels in trophoblast cell lines differ from those in the *in vivo* placenta (Karahoda et al., 2021). Similarly, cytochrome P450 enzymes, including CYP2C8, CYP2C9, and CYP2J2, are poorly expressed (Kammala et al., 2022). Second, the expression or localization of placental cellular transporters may also vary with temporal placental development; thus, the cell model cannot fully replicate the dynamic changes in transporter expression that occur during different gestational stages (Veiga-Lopez et al., 2025). Future research should focus on three key directions to enhance BeWo-based placental model characteristics (Be et al., 2025): developing microfluidic co-culture systems integrating BeWo cells with human umbilical vein endothelial cells (HUVECs) (Fuenzalida et al., 2024) under physiological shear stress; (Lupattelli et al., 2014) applying CRISPR editing to introduce disease-specific mutations; (Subramanian et al., 2023) establishing 3D organoid models through BeWo-fibroblast co-culture. These advanced models require standardized validation, including cAMP-induced syncytialization, TEER monitoring, and transporter functionality assays, to ensure regulatory acceptance and enhance the predictive accuracy in placental drug-transport studies.

### 3.2 Caco-2 cell

The Caco-2 cell model is widely used to study drug permeability (Artursson et al., 2001). Derived from human colon cancer cells, Caco-2 cells differentiate into polarized monolayers that resemble small intestinal epithelial cells and mimic the barrier function of the human intestine (Sambuy et al., 2005). Over the past 2 decades, the Caco-2 monolayer model has become a crucial tool for preclinical drug development, particularly for permeability screening (Panse and Gerk, 2022). The Caco-2 monolayer allows the study of all major absorption pathways, such as passive transcellular and paracellular diffusion and carrier-mediated active and facilitated transport (Volpe, 2020).

Caco-2 cells form a continuous monolayer barrier for intestinal barrier simulation, and similarly, researchers have adapted this well-characterized system as a functional surrogate for placental barrier studies, leveraging its reproducible tight junction formation and transport properties. Mian et al. utilized a confluent Caco-2 monolayer system in Transwell chambers, with pre-experimental TEER measurements confirming barrier integrity (Mian et al., 2020). This established model served as an effective placental barrier surrogate for characterizing acetaminophen transport and determining the apparent permeability coefficient (Papp) across the placental barrier. This experimental approach has been similarly applied to evaluate the transplacental permeability of various therapeutic agents, including nifedipine and vancomycin (Werdan Romão et al., 2024; Yan et al., 2024) (Table 1).

The utility of Caco-2 cells as an *in vitro* model for placental transport research is constrained by several inherent biological and methodological limitations. For instance, there are biological differences between Caco-2 cells and placental epithelial cells, which may lead to overestimation or underestimation of drug permeability. Caco-2 cells express key efflux transporters found in the STB, including P-glycoprotein (P-gp/ABCB1), BCRP (ABCG2), and certain MRPs (ABCC family) (Bai et al., 2016), but they show limited expression of uptake transporters such as OCTs and OATs (Wenzel et al., 2021). Therefore, Caco-2 cells are suitable for studying efflux-mediated transport but have limitations in modeling placental uptake processes. To bridge physiological gaps between models and human placenta, researchers employ genetic engineering strategies to upregulate key protein expression (Ohta et al., 2020) and apply biosimilar mucus to mimic native mucosal barriers (Panse and Gerk, 2022). Nevertheless, such models provide valuable preliminary data when access to more physiologically relevant systems is limited.

### 3.3 Primary trophoblasts

Primary cytotrophoblasts (CTBs) isolated from term human placentas that spontaneously fuse and differentiate into STB (Li and Schust, 2015)-like cells *in vitro* are used to investigate the functions of the STBs (Hawkins et al., 2025) and placentas with multiple modifications (Motomura et al., 2025). These cells maintain native transporter expression profiles (e.g., BCRP and P-gp) and metabolic activity, which are the most physiologically relevant *in vitro* cell model for studying human placental function, but unfortunately, CTBs are rarely used for placental barrier-related studies. In addition, trophoblast stem cells (hTSCs) were derived from human villous CTBs and human blastocysts (Okae et al., 2018). These long-term stem cell cultures may give rise to CTBs, EVTBs, and STB-like cells that show transcriptomes similar to those of the corresponding primary trophoblasts (Shannon et al., 2024), which have emerged as a powerful tool for modeling the placental cytotrophoblast *in vitro* (Karakis et al., 2025). Given that the primary focus of our investigation centers on placental barrier function, a comprehensive discussion of this aspect falls beyond the scope of this study.

## 4 Organ experimental models for placental transfer

*Ex vivo* placental perfusion experiments, first proposed by Professor Maurice Panigel in 1967, have been continuously refined to address challenges such as revascularization and placental hypoxia resistance (Schneider et al., 2021). As research has expanded in recent years, new methods of studying placental drug-transport have emerged. Organ-on-a-chip technology, which integrates microfluidics and cell culture, replicates the human organ microenvironment on a chip and accurately simulates the blood flow, oxygen exchange, and nutrient delivery.

### 4.1 *Ex vivo* placental perfusion experiments

*Ex vivo* placental perfusion experiments are crucial *ex vivo* models used to study the transport of exogenous substances and the effects of hormones (Page, 1991). This model simulates the exchange of substances between the mother and fetus by creating specific external conditions and perfusion systems that closely mimic physiological processes in the human body. This model allows researchers to study the mechanisms of exogenous substance transfer, fetal exposure, and associated diseases (Villalobos-Labra et al., 2023). In addition, this model can be used to study the effects of factors such as inflammation and oxidative stress on biochemical and physiological imbalances that are closely linked to conditions such as preeclampsia, fetal growth restriction, diabetes, and microbial infections (Koren and Ornoy, 2018).

**FIGURE 2:**
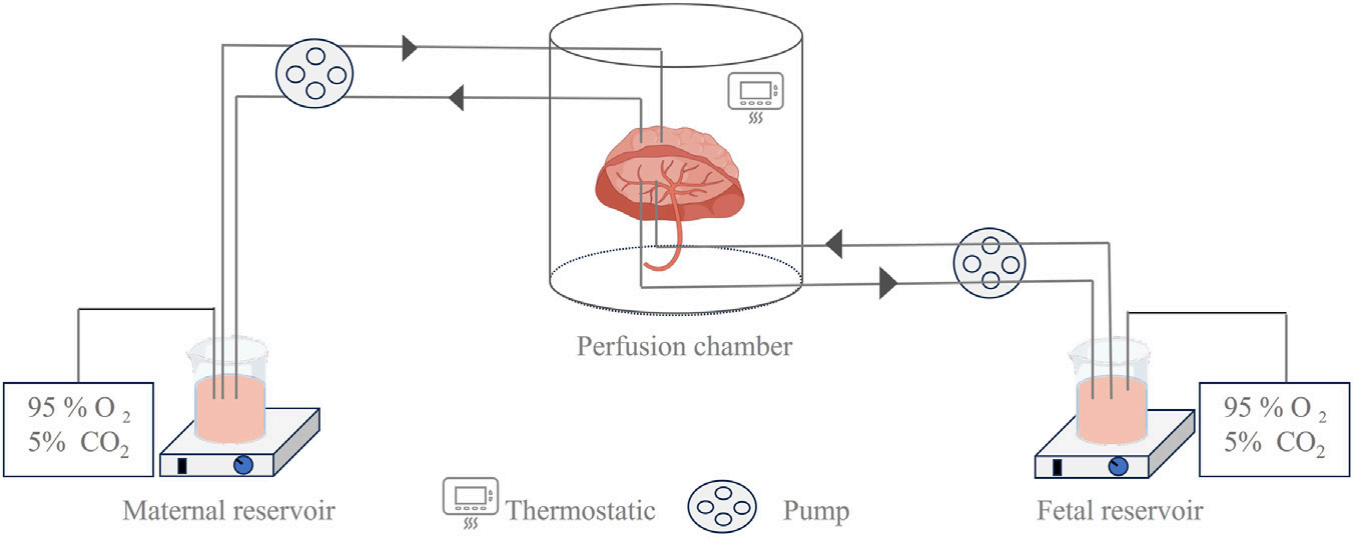
Illustration of the *ex vivo* dual-side cotyledon perfusion model. This device shows a schematic diagram of a dual closed-circuit extracorporeal placental perfusion test, which more closely simulates the process of maternal–fetal substance exchange and is widely used in the study of maternal–fetal transport mechanisms.

*Ex vivo* placental perfusion experiments comprised maternal and fetal reservoirs, the placenta, and a perfusion pump that simulated blood flow with controlled pressure and flow rate (Figure 2). A well-defined artery–vein pair on the fetal-side cotyledon with minimal branching was chosen for cannulation following the confirmation of intact placental integrity post-delivery (Jovelet et al., 2015). The system only remains patent for 2–6 h, so only short-term assessments can be made, and the circuit design (open, semi-closed, or closed loop) determines the physiological relevance, ranging from first-pass transfer simulation to steady-state PK assessment. The transfer process is mathematically described by Fick’s first law using the transfer equations outlined by Mian et al. (2020). Our review focused on 28 recent studies (2015–2025) employing *ex vivo* human placental perfusion to investigate transplacental kinetics of various therapeutics, including antivirals, psychotropics, and biologics (Table 2). This 10-year timeframe was selected to account for methodological advancements while ensuring data comparability. Notably, researchers exhibit a distinct preference for double-closed perfusion systems, which consistently operate for significantly longer durations than open-circuit configurations (avg. 3.6 h vs. 1.9 h). This operational divergence substantiates the superior suitability of double-closed systems for steady-state PK investigations. Small molecules dominate the studied drugs (30/34, 88%), exposing a critical evidence gap for biologics/antibodies despite their clinical relevance.

**TABLE 2:** Overview of studies that reported *ex vivo* placental perfusion models.

Drug	Dosage	Perfusion system	Duration	FTR	CLI	CAI	TPTs/SF	Ref.
Acetaminophen	1,000 mg	Double-closed	3.5 h	0.737	36 mL/min	NA	NA	Mian et al. (2020)
Aldosterone	1.5 × 10^−9^ M; 1.5 × 10^−7^ M	Open-circuit	2 h	NA	NA	NA	TNF-α decreased; PlGF increased	Mistry et al. (2022)
Bictegravir and cabotegravir	550 ng/mL; 1,650 ng/mL	Open-circuit	1.5 h	7%, 10%	22% and 21%	NA	NA	Pencolé et al. (2020)
Bromocriptine	10 ng/mL (low); 100 ng/mL (high)	Double-closed	3 h	0·37 ± 0·03 (low); 0·37 ± 0·03 (high)	NA	NA	NA	Zheng et al. (2019)
Ciprofloxacin	1.6 μg/mL	Double-closed	3 h	0.53	0.28 mL/min	22%	NA	Noergaard et al. (2021)
CZP	200 μg/mL	Double-closed	6 h	Undetected	NA	NA	CZP: FcRn non-binder	Porter et al. (2016)
Doravirine	0.96 mg/L	Double-closed	3 h	82%	37.2 mL/min	NA	NA	Bukkems et al. (2022)
Dolutegravir	4 μg/mL	closed-open	3 h	0.57–1.51	1.03 ± 0.06 mL/min	NA	NA	Freriksen et al. (2020)
Dolutegravir	4.2 mg/L	Double-closed	3 h	0.6 ± 0.2	NA	NA	NA	Schalkwijk et al. (2016)
EMPA, DAPA, and CANA	150 μg/L	Double-closed	6 h	0.38, 0.67, 0.62	NA	NA	NA	Kuoni et al. (2024)
Etanercept and infliximab	100 μg/mL	Double-closed	6 h	0.04, 1.6 ± 0.4	NA	NA	NA	Eliesen et al. (2020)
Fexofenadine	75 ng/mL	Double-closed	1.5 h	0.17–0.19	NA	NA	NA	Pinto et al. (2021)
Gefitinib, Imatinib, and Erlotinib	500, 1,000, 1,500 ng/mL	Open-circuit	1.5 h	16.8%, 10.6%, and 31.4%	0.59 ± 0.04, 0.48 ± 0.14, and 0.93 ± 0.03	NA	NA	Jovelet et al. (2015)
Lacosamide	2.5 μg/mL; 10 μg/mL	Double-closed	3 h	0.94, 0.86	NA	NA	SLC19A1 decreased	Berman et al. (2022)
LMV, MBV	480 mg and 400 mg	Open-circuit	1.5 h	0.09 ± 0.01,0.1 ± 0.01	0.35 ± 0.06 and 0.5 ± 0.07	0.04 ± 0.02, 0.04 ± 0.01	NA	Faure Bardon et al. (2020)
Omalizumab	28.6 μg/mL; 45.8 μg/mL; 65.7 μg/mL; 107.0 μg/mL	Double-closed	4 h	0.02%–0.03%	NA	NA	PEA: doubled transfer; IgE: unchanged	Kothari et al. (2022)
OP, OC	65.2 ng/mL, 348 ng/mL	Double-closed	3 h	12.39% ± 3.26% (OC) and 10.17% ± 2.03% (OP)	0.36 ± 0.11 and 0.29 ± 0.06	NA	NA	Huang et al. (2017)
Oxytocin	0.8 ng/mL	Double-closed	3 h	15% (0 min) → 173% (180 min), equilibrium at 90 min	NA	NA	NA	Nathan et al. (2021)
Pentoxifylline	10 mg/L	Double-closed	3 h	0.9–1.0	NA	NA	NA	Broekhuizen et al. (2022)
Pravastatin	0.2 μmol/L	Double-closed	5 h	NA	NA	NA	PlGF/sFlt-1/sEng/p-eNOS: unaltered	B et al. (2017)
Rivaroxaban	250 ng/mL	Double-closed	3 h	0.69	NA	NA	NA	Bapat et al. (2015)
Rmd, GS	4,000 ng/mL	Double-closed	3 h	8.15% and 9.9%	NA	NA	NA	Louchet et al. (2023)
Sildenafil	500 ng/mL	Double-closed	3 h	0.37 ± 0·03 (health); 0.47-0·66 (PE)	NA	NA	Normal: PDE5, eNOS, iNOS; decreased in PE:PDE1	Hitzerd et al. (2019)
Vancomycin	NA	Double-closed	NA	NA	0.002421 L/h/mL	NA	NA	Yan et al. (2024)
Venlafaxine	18.1 ± 2.1 μg/L	Double-closed	3 h	0.89	NA	22%	NA	Hirschmugl and Wadsack (2022)

Abbreviations: FTR, fetal–maternal ratio; CLI, clearance index; CAI, cotyledon accumulation index; TPTs, placental transporter proteins; SF, secreted factor; TNF-α, tumor necrosis factor-alpha; PlGF, placental growth factor; EMPA, empagliflozin; DAPA, dapagliflozin; CANA, canagliflozin; SGLT2-i, sodium glucose co-transporter 2 inhibitors; LMV, letermovir; MBV, maribavir; PEA, peanut allergen; EC50, half-maximal effective concentration; Rmd, remdesivir; GS, GS‐441524; PE, preeclampsia; OP, oseltamivir phosphate; OC, oseltamivir carboxylate, active metabolite of oseltamivir phosphate; sFlt-1, soluble fms-like tyrosine kinase-1; sEng, soluble endoglin; p-eNOS, endothelial nitric oxide synthase phosphorylation; CZP, certolizumab pegol; PDE1, phosphodiesterase-1; PDE5, phosphodiesterase-5; iNOS, inducible nitric oxide synthase; LLOQ, lower limit of quantification; NA, not applicable.

In addition, *ex vivo* placental perfusion models have emerged as pivotal tools for evaluating nanocarrier transplacental behavior, with recent studies demonstrating that targeted lipid nanoparticles (LNPs) remain largely confined to maternal circulation, exhibiting minimal fetal transfer, thereby suggesting their utility in preventing unintended drug passage across the placental barrier (van Kammen et al., 2024). Complementing these findings, a review of 16 perfusion studies further elucidates that nanoparticle (NP) transport efficiency is critically governed by physicochemical determinants, particularly sub-100-nm dimensions and surface modifications such as PEGylation, which collectively modulate placental uptake kinetics (Aengenheister et al., 2021). These insights establish that rational nanocarrier design represents a promising strategy for blocking unintended fetal drug exposure.

Despite being non-invasive, *ex vivo* placental perfusion experiments are constrained by the limited availability of human placental tissue (Parks, 2015) and the need to maintain strict physiological conditions (37 °C, fetal arterial pressure 30 mmHg–60 mmHg, 95% O_2_/5% CO_2_ (maternal), 95% N_2_/5% CO_2_ (fetal), oxygen consumption, glucose, lactate, and HCG indicators) (Schneider, 2015). Furthermore, the intricate vascular network of the placenta makes it challenging to accurately replicate blood flow and material exchange *ex vivo*, resulting in a low success rate and limiting its application (Mathiesen et al., 2010). Unfortunately, full-term placental extracts may not reflect the evolution of the placenta during pregnancy.

Future advancements should refine placental perfusion systems by implementing multi-cannula designs (e.g., 22 maternal cannulas) to replicate physiological oxygen gradients, which are confirmed by direct measurements to maintain the target median oxygen tension (∼50 mmHg) and reduce hypoxic heterogeneity in the intervillous space (Schneider, 2015). Concurrently, preserving endothelial integrity requires real-time monitoring of trophoblastic vacuolization, which is a histopathological marker strongly correlated with fetal circuit leakage, combined with mechanical stress reduction via low-pressure perfusion (<60 mmHg) and albumin-supplemented media (Maroun et al., 2014). Crucially, mitigating oxidative stress demands protocol modifications, such as pre-perfusion hypothermia (4 °C) and HO-1 inducers (e.g., hemin) to activate antioxidant pathways, pulsed reoxygenation to minimize ROS bursts, and antibiotic supplementation to prevent bacterial-induced inflammation (Schneider, 2009). Moreover, the standardization of placental perfusion techniques should be accelerated to ensure data consistency across research centers, facilitate cross-study validation, and promote broader adoption (Schneider et al., 2022). It is foreseeable that with continuous optimization, placental perfusion experiments will hold significant application in placental PK research.

### 4.2 Organ-on-a-chip

**FIGURE 3:**
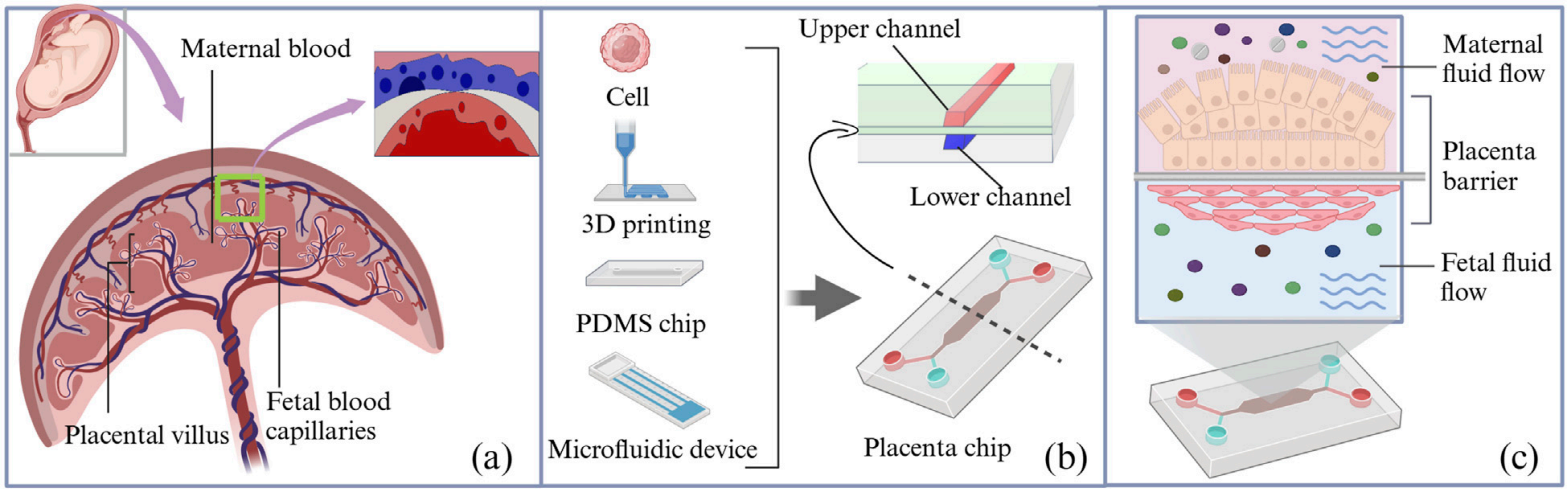
Schematic diagram of the structure of the placenta organ-on-a-chip device. **(a)** Schematic diagram of the placental barrier. **(b)** Schematic of composition and cross-sectional view of the organ-on-a-chip microfluidic device; a porous membrane separates the maternal and fetal channels in the microfluidic device. **(c)** The maternal and fetal sides were composed of BeWo cells and HUVECs, respectively. PDMS, polydimethylsiloxane.

An organ-on-a-chip is an *in vitro* biomimetic microfluidic device that replicates the structure and function of human organs, typically at 10^–6^ to 10^–4^ times the size of the actual organ (Shuler, 2017). It provides a model that closely simulates the *in vivo* cellular environment, enabling cells and tissues to grow and complete their life cycles. This device uses microchip technology to create microfluidic cell culture chambers, where live cells are organized according to the organ physiology. By reproducing multicellular structures, tissue interfaces, and vascular perfusion, it mimics *in vivo* organ function and cellular environments (Figure 3) (Cavero et al., 2019). The basic organ-on-a-chip is fabricated using standard soft-lithography techniques, where a 10:1 mixture of polydimethylsiloxane (PDMS) substrate and curing agent is cast onto an SU-8 master mold to form a bilayer or multicavity microfluidic structure with precisely defined channels. A porous polyester track-etched membrane or polycarbonate membrane is then sandwiched between the microchannels as a biological barrier, which is subsequently assembled and selectively coated with extracellular matrix components to form a biomimetic placental barrier through plasma bonding.

A comprehensive literature search revealed that, to date, placental drug transport has been evaluated and reported for only five pharmacological agents using the placenta-on-a-chip technology under controlled microphysiological conditions (Table 3), involving opioids, hypoglycemic agents, statins, and other drugs. Evidently, placenta-on-a-chip studies commonly use a core co-culture model of paired trophoblast cells (BeWo/STB) and endothelial cells (HUVECs/HPVECs) to replicate the maternal–fetal barrier. For statin (e.g., rosuvastatin)-specific studies, this basis can be extended to complex multicompartmental systems (3 layers–7 layers), which include fetal membrane cells (AECs, AMCs, and CTCs) and decidual cells (DCs) to mimic tissue interactions. Barrier integrity is generally verified by a tripartite protocol: immunostaining, FITC-dextran permeability assay, and morphological analysis, while some studies are supplemented with TEER measurements. Functionally, the simpler two-chamber design is sufficient for passive diffusion studies, whereas the ≥4-chamber configuration becomes crucial for drug metabolism studies, allowing important analyses of statin-derived metabolites and inflammatory cascades.

**TABLE 3:** Overview of reported organ-on-a-chip studies on placental drug transfer.

Drug/compound	Dosage	Duration	Cell line (maternal)	Cell line (fetal)	Chambers	Barrier integrity and function verification	Key findings	Ref.
Naltrexone/6ß-naltrexol	100 ng/mL	8 h	BeWo cells	HUVECs	2	Immunostaining FITC-dextran flux Microvilli imaging	FTR: 2.5% (naltrexone), 2.2% (6β-naltrexone)	Pemathilaka et al. (2022)
Caffeine	0.25 mg/mL	7.5 h	BeWo cells	HUVECs	2	Immunostaining FITC-dextran flux Microvilli imaging	FTR: 1.3% (at 5 h)	Pemathilaka et al. (2019b)
Glyburide	1 × 10^−6^ M	7.5 h	STB	HPVECs	2	Immunostaining TEER measurement FITC-dextran flux	FTR: 5.6%; clearance: BCRP-mediated exocytosis	Blundell et al. (2018)
Pravastatin/rosuvastatin	200 ng/mL	24 h	DCs	Fetal membrane (AECs, AMCs, and CTCs); Placenta (BeWo, STB, and HUVECs)	3,4	Immunostaining FITC-dextran flux Cell morphology and coverage check Cytotoxicity assay Cytokine analysis	Metabolites produced; anti-inflammatory cytokines increased	Richardson et al. (2022)
Pravastatin	200 ng/mL	24 h	DCs, STB, BeWo	CTCs, AMCs, AECs, and HUVECs	7	Immunostaining FITC-dextran flux Cell morphology and coverage check Cell viability assay	FTR: 18%	Kammala et al. (2023)
Rosuvastatin	200 ng/mL		BeWo cell	AECs, AMCs, and CTCs	4	Immunohistochemistry Flow cytometry	OATP2B1: mediate transplacental transport	Ganguly et al. (2021)

Abbreviations: HUVECs, human umbilical vein endothelial cell lines; TEER, trans-epithelial electrical resistance; HPVECs, human placental villous endothelial cells; FTR: fetal-to-maternal ratio; BCRP, breast cancer resistance protein; AECs, amnion epithelial cells; AMCs, amnion mesenchymal cells; CTCs, chorionic trophoblast cells; DCs, decidual cells; STB, syncytiotrophoblasts, BeWo + forskolin.

The development of three-dimensional (3D) placenta-on-a-chip models has been accelerated by the availability of microfluidic devices, commercial chips, and 3D bioprinting technologies. For instance, HUVECs and placental BeWo cells were co-cultured (1:3) for the first time to form spheroids (microtissues) on a 3D Petri Dish^®^ mold, which mimicked *in vivo* responses more closely than 2D systems, in three main ways (Be et al., 2025): spontaneous self-organization into a vascularized histoarchitecture with HUVECs peripherally enveloping a BeWo core, mimicking placental villi (Lupattelli et al., 2014); enhanced intercellular junction formation (E-cadherin/ZO-1) and cytoskeletal reorganization yielding physiologically relevant TEER values (Subramanian et al., 2023); superior functional maturation evidenced by elevated β-hCG secretion and drug metabolic capacity that more accurately predicted *in vivo* pharmacological responses (Öztürk et al., 2024). Cao et al. developed a biomimetic placental barrier model that incorporated glucose transport and hCG secretion (Cao et al., 2023). Another study developed a second-trimester (14 W–28 W) placental model (Vidal et al., 2024) that offered valuable insights into placental drug transfer. These methodological advancements, when successfully implemented in transplacental drug studies, are anticipated to significantly enhance the quality of evidence regarding fetal drug exposure in future research endeavors.

The placenta-on-a-chip technology offers significant advantages over traditional trophoblast monolayer models by recreating the multilayer structure of the placental barrier and the hemodynamic environment (Zambuto et al., 2024). However, the model has several limitations. First, current microarrays struggle to replicate complex overall physiological environments, which hamper their ability to model systemic diseases. Second, current placenta-on-a-chip models are a poor representation of the human placental villous structure. Another limitation is that as the complexity of 3D co-culture models increases, it becomes more challenging to perform high-resolution screening and accurately localize the tissues (Bhatia and Ingber, 2014). Future placenta-on-a-chip development must, therefore, improve both physiological fidelity and microscale spatial resolution (Pemathilaka et al., 2019a; Esch et al., 2015), while incorporating gestational changes (Blundell et al., 2018). Critical advancements must target the following (Be et al., 2025): multi-lineage co-culture optimization (trophoblast/endothelial/stromal cell interactions) to maintain tissue-relevant cellular diversity (Lupattelli et al., 2014); sub-10 μm spatial resolution achievement through nanopatterned surface functionalization (Subramanian et al., 2023); hierarchical biomaterial development integrating decellularized ECM and precision-bioprinted villous structures; (Phillips et al., 2024) programmable microenvironment platforms with feedback-controlled gas/endocrine factor delivery, enabling phase-transition modeling between gestational periods. Following significant advancements in organ-on-a-chip technology, subsequent research efforts should prioritize the implementation of these systems for longitudinal exposure studies and polypharmacy interaction analyses that are specifically designed to characterize the teratogenic consequences of sustained drug administration during critical periods of fetal development.

## 5 *In silico* simulation for placental transfer

Animal studies remain difficult to interpret due to structural and functional inter-species placental differences. The *ex vivo* perfusion of the human placental cotyledon is the method of reference for studying the human placental transfer of drugs because it is thought to mimic the functional placental tissue. However, it is challenged by the constraints of limited placental tissue and low experimental success rates. Recently, *in silico* techniques have further been advanced as complementary tools to validate experimental placental transfer data, offering a promising alternative for high-throughput screening of potential fetotoxicity at the early stages of drug design. PBPK models are promising *in silico* simulation tools that provide valuable insights into drug PK, particularly those that are difficult to assess *in vivo*. Several other methods have also been used to investigate placental PK.

### 5.1 Physiologically based pharmacokinetics (PBPK)

**FIGURE 4:**
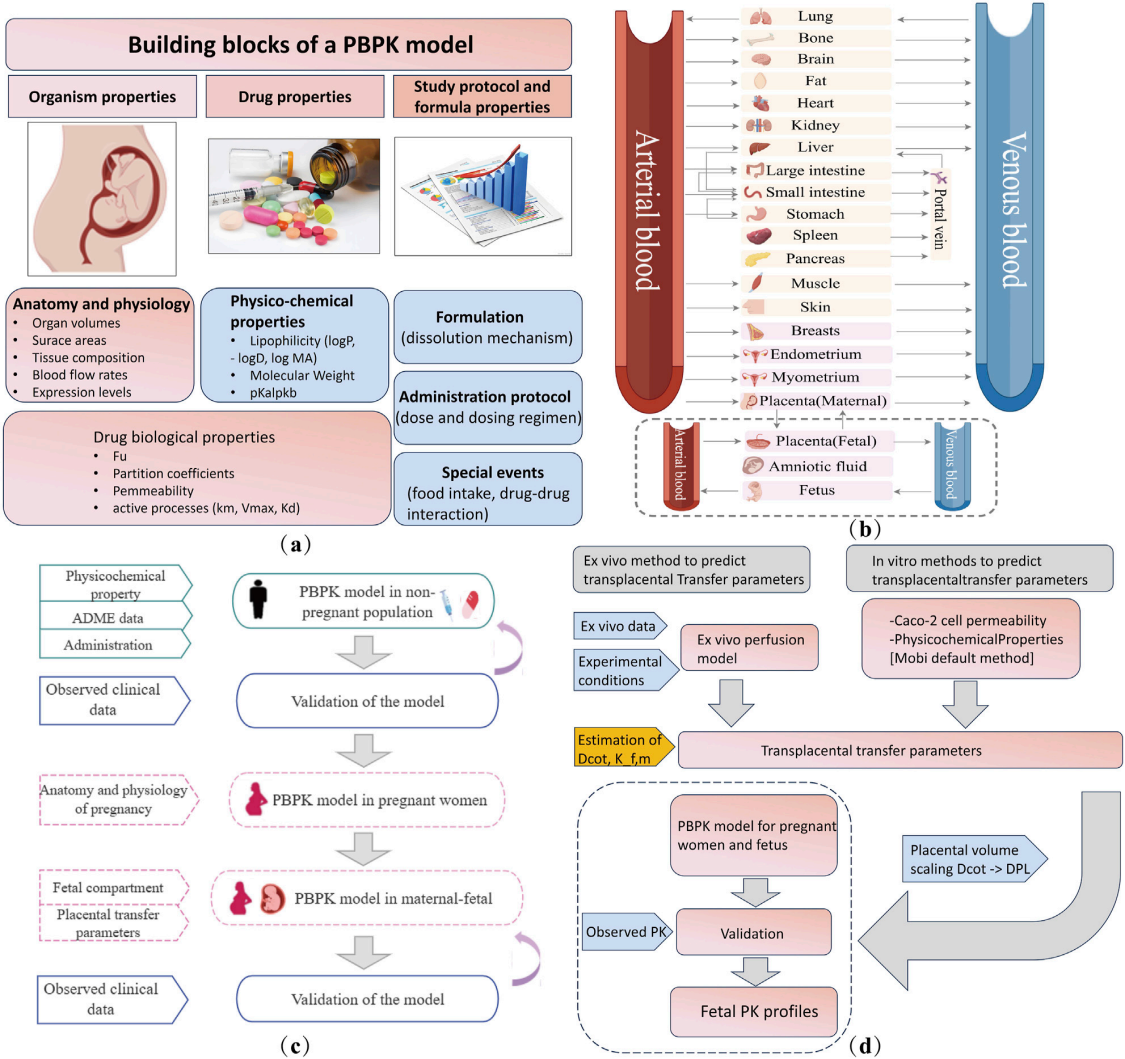
Maternal–fetal PBPK model details. **(a)** Building blocks for developing a PBPK model. **(b)** Structural diagram of the PBPK model of a pregnant woman in 27 compartments. The pink block section shows nine pregnancy-specific compartments. Fetal compartments can be refined according to the study objectives. **(c)** Workflow of maternal–fetal PBPK model development and evaluation. **(d)** Transplacental parameter estimation and integration. ADME, absorption, distribution, metabolism, and excretion. Dcot, transcotyledon passive diffusion clearance; Kf,m, partition coefficient between the fetal and maternal compartment; Dpl, transplacental passive diffusion clearance; PK, pharmacokinetics.

The PBPK model uses mathematical simulations to describe the absorption, distribution, metabolism, and excretion (ADME) of drugs by integrating the anatomical, physiological, and drug-specific data with preclinical and clinical information to simulate PK (Dallmann et al., 2018). When applied to special populations, such as pregnant women and neonates, PBPK models incorporate organ development and individual variations. The key components of PBPK model development are shown in Figure 4a. The general workflow for developing a maternal–fetal PBPK model is shown (Figure 4c). First, a non-pregnant PBPK model was established and validated by comparison with the observed *in vivo* data. Once the non-pregnant PBPK model accurately captured PK, all drug-specific parameters were fixed. Pregnancy-specific changes [e.g., increase in blood volume, changes in metabolism, increased cardiac output, lower hypertension, increased renal blood flow, and variation with gestational week (Zhang et al., 2017; Zhang and Unadkat, 2017)] were then incorporated to extend the model to a pregnancy-specific version (Dallmann et al., 2017), which involved adding pregnancy-specific compartments to the 27-compartment model (Figure 4b). The maternal–fetal PBPK (M–F PBPK) model is linked via a placental permeability-limited model subdivided into maternal blood, placental tissue, and fetal blood compartments (Zhang and Unadkat, 2017). Transplacental clearance (Yan et al., 2024) and other key parameters were assessed using various methods (Figure 4d) and were included in the final formation of the M–F PBPK models. In addition, depending on the purpose of the study, researchers may selectively incorporate placental drug metabolism/efflux transporters into the model.

**FIGURE 5:**
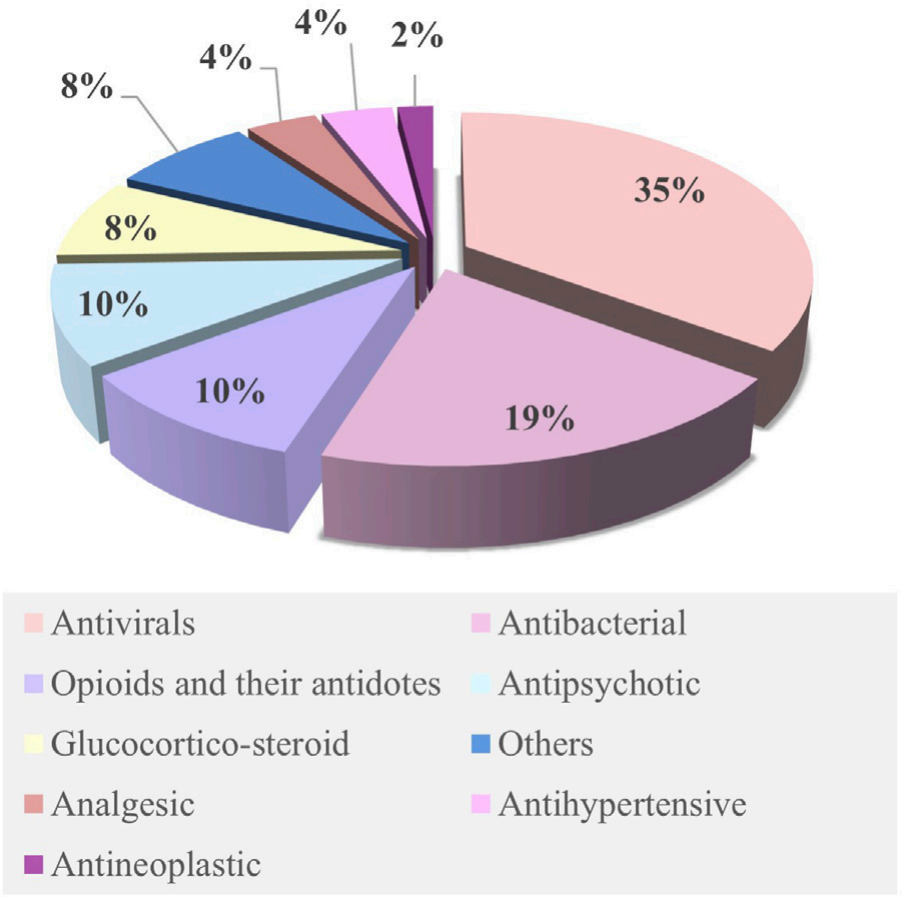
Ratios of various drugs studied in the M–F PBPK model. Publications focusing on M–F PBPK were identified by systematically searching the scientific literature for peer-reviewed articles using PubMed. Relevant key terms were used to identify publications (e.g., “Physiologically based pharmacokinetic model,” “Pregnancy,” and “Foetal”). No date restriction was set. Collected publications were scrutinized for possible additional references not covered by this search.

The M–F PBPK model was developed in 2015 (Dallmann et al., 2018) and has rapidly advanced since 2020. Some research groups have extensively worked on PBPK models to predict fetal drug exposure and showed very encouraging results. In Table 4, we summarize the developed M–F PBPK models encompassing 42 pharmacological agents from 39 studies, including antiviral agents, antibacterial, opioids, antipsychotics, hormones, analgesics, anti-hypertensives, and antineoplastic agents. The dominance of antivirals (39%) in all M–F PBPK studies is highlighted in Figure 5, which shows that the use of antiviral drugs in the treatment of HIV/AIDS and hepatitis B is a global concern. During our literature search, we found several interesting research areas, including PBPK models that focused on predicting maternal drug concentrations and fetal environmental toxin exposure. However, these topics were not the focus of this study and have not been discussed further.

**TABLE 4:** Overview of reported fetal drug exposure studies for M–F PBPK (categorized by the pharmacological effect).

Compound	Administration	Software	Placental permeability	No. of compartments	Placenta compartments	Fetal Compartments	Validation	Clinical translation	Ref.
Analgesic									
Acetaminophen	po	PK-Sim /MoBi	*Ex vivo* perfusion model, Caco-2 assay, and physicochemical properties	28	P-m and P-f	AB, VB, AF, fetal liver, and RoB	MP, UCP	No dosage adjustment required	Mian et al. (2020)
Indomethacin	po	Simcyp	Published data	34	P-m and P-f	Full fetal PBPK model	MP, UCP	Fetal exposure risks elevated in third trimesters	Pillai et al. (2022)
Antibacterial									
Cefazolin	iv	GastroPlus	Parameter identification	NA	P-m and P-f	AB, VB, AF, and fetal body	MP, UCP, and AF	NA	Szeto et al. (2021)
Cefuroxime	iv. or im	GastroPlus	Parameter identification	NA	P-m and P-f	AB, VB, AF, and fetal body	MP, UCP, AF	NA	Szeto et al. (2021)
Cefazolin, Cefuroxime, Amoxicillin	iv. or im. or iv. bolus	Simcyp V 20	Published data	35	P-m, P-t, and P-f	Full fetal PBPK model	MP, UCP, and AF	No dosage adjustment required	Abduljalil et al. (2022a)
Ampicillin	iv	Simcyp V 21	Physicochemical properties	35	P-m, P-t, and P-f	Full fetal PBPK model	MP, UCP	No dosage adjustment required	Li and Xie (2023)
Cefuroxime	iv	PK-Sim /MoBi	Parameter identification	41	P-m, P-f	AB, VB, AF, brain, fat tissue, heart, liver, lungs, kidneys, gastrointestinal tract, muscles, and RoB	MP, UCP, AF, heart, liver, and lungs	NA	Liu et al. (2024)
Vancomycin	iv	Simcyp	*Ex vivo* perfusion model, QSPR model, and Caco-2 assay	35	P-m, P-t, P-f	Full fetal PBPK model	MP, UCP	NA	Yan et al. (2024)
Ceftazidime	iv. or im	Simcyp V 23	Physicochemical properties	35	P-m, P-t, P-f	Full fetal PBPK model	MP, UCP, and AF	NA	Abduljalil et al. (2024)
Metronidazole	Iv. or po	GastroPlus	Published data	NA	Not reported	Not reported	MP, UCP	NA	Le Merdy et al. (2024)
Antipsychotic									
Oxcarbazepine	po	PK-Sim /MoBi	*Ex vivo* perfusion model, Caco-2 assay, physicochemical properties	27	P-m, P-f	AB, VB, AF, and fetal body	MP, UCP	Dose ≥1.5× baseline in 2nd/3rd trimesters	He et al. (2022)
Levetiracetam	iv. or po	PK-Sim /MoBi	Physicochemical properties	27	P-m, P-f	AB, VB, AF, and fetal body	MP, UCP	Dose: 1.2×, 1.6×, 1.5× baseline in 1st/2nd/3rd trimesters	Chen et al. (2023)
Sertraline	po	R Studio	Published data	15	P-m, Pf	AF, fetal blood, gut, liver, lung, and RoB	MP, UCP	NA	Lartey et al. (2023)
Midazolam	iv. or po	GastroPlus	Published data	NA	Not Reported	Not reported	MP, UCP	NA	Le Merdy et al. (2024)
Carbamazepine	po	PK-Sim/Mobi	*Ex vivo* perfusion model, Caco-2 assay, and physicochemical properties	27	P-m, P-f	AB, VB, AF, and fetal body	MP, UCP	Dose: 1.4×, 1.8×, 2.1× baseline in 1st/2nd/3rd trimesters	Chen et al. (2024)
Antivirals									
Darunavir	po	Berkeley Madonna/Simcyp	*Ex vivo* perfusion model	13	Placenta	AF, fetal blood, and fetal body	MP, UCP, AF	No dosage adjustment required	Schalkwijk et al. (2018)
Dolutegravir	po	Berkeley Madonna/Simcyp	*Ex vivo* perfusion model	13	Placenta	AF, fetal blood, and fetal body	MP, UCP, AF	No dosage adjustment required	Freriksen et al. (2020)
Dolutegravir	po	PK-Sim/MoBi	Published data	27	P-m, P-f	AB, VB, AF, and fetal body	MP, UCP, NP	NA	Liu et al. (2021), Liu et al. (2020a)
Dolutegravir	po	MATLAB	Published data	19	Placenta	AB, VB, AF, brain, kidney, liver, and RoB	MP, NP	No dosage adjustment required	Shenkoya et al. (2021)
Efavirenz	po	MATLAB	Published data	19	Placenta	AB, VB, AF, brain, kidney, liver, and RoB	MP, NP	NA	Shenkoya et al. (2021), Atoyebi et al. (2019)
Emtricitabine	iv. or po	Simcyp/R	*Ex vivo* perfusion model	20	P-m, P-f	AF, fetal blood, and fetal body	MP, UCP	NA	De Sousa Mendes et al. (2016)
Emtricitabine	po	PK-Sim/MoBi	*Ex vivo* perfusion model	27	P-m, P-f	AB, VB, AF, and fetal body	MP, UCP, NP	NA	Liu et al. (2020b)
Acyclovir	iv. or po	PK-Sim/MoBi	Parameter identification	27	P-m, P-f	AB, VB, AF, and fetal body	MP, UCP	NA	Liu et al. (2020b)
Nevirapine	iv	R	*Ex vivo* perfusion model	20	P-m, P-f	AF, fetal blood, and fetal body	MP, UCP	No dosage adjustment required	De Sousa Mendes et al. (2017)
Raltegravir	po	PK-Sim/MoBi	*Ex vivo* perfusion model	27	P-m, P-f	AB, VB, AF, and fetal body	MP, UCP, NP	NA	Liu et al. (2020a)
Tenofovir	iv. or po	Simcyp/R	*Ex vivo* perfusion model	20	P-m, P-f	AF, fetal blood, and fetal body	MP, UCP, AF	NA	De Sousa Mendes et al. (2016)
Zidovudine	iv. or po	MATLAB/Simcyp	Published data	12	P-m, P-t, and P-f	AB, VB, AF, brain, gut, kidney, liver, and RoB	MP, UCP	NA	Zhang et al. (2017), Zhang and Unadkat (2017)
Acyclovir, Emtricitabine, Lamivudine	iv. or po	Simcyp V 20	Published data	35	P-m, P-t, and P-f	Full fetal PBPK model	MP, UCP, and AF	NA	Abduljalil et al. (2022b)
Doravirine	iv. or po	Simcyp	*Ex vivo* perfusion model	31	P-m, P-t, and P-f	Full fetal PBPK model	MP, UCP	Increase in dosage, unspecified	Bukkems et al. (2022)
Cabotegravir, Rilpivirine	im	SimBiology /MATLAB	Caco-2 assay, physicochemical properties	30	P-m, P-f	AB, VB, and fetal body	MP, UCP	Cabotegravir: no dosage adjustment required; rilpivirine: increase in dosage, unspecified	Atoyebi et al. (2024)
Dolutegravir	po	Simcyp	*Ex vivo* perfusion model	12	P-m, P-t, and P-f	AB, VB, AF, brain, gut, kidney, liver, and RoB	MP, UCP	No dosage adjustment required	Ning et al. (2024)
Antihypertensive									
Nifedipine	oral	Simcyp V 22	Caco-2 assay	35	P-m, P-t, and P-f	Full fetal PBPK model	MP, UCP, AF	Increase in dosage, unspecified	Werdan Romão et al. (2024)
Metoprolol	iv. or po	GastroPlus	Published data	NA	Not Reported	Not Reported	MP, UCP	NA	Le Merdy et al. (2024)
Antineoplastic									
Crizotinib	po	Simcyp/MATLAB	PBMP model	12	P-m, P-t, and P-f	AB, VB, AF, brain, gut, kidney, liver, and RoB	MP, UCP, and placenta tissue	NA	Roelofsen et al. (2022)
Hormones									
BET, DEX	im	Simcyp/MATLAB	Caco-2 assay	12	P-m, P-t, and P-f	AB, VB, AF, brain, gut, kidney, liver, and RoB	MP, UCP	NA	Anoshchenko et al. (2021)
BET, DEX	iv. or im. or po	Simcyp/MATLAB	*Ex vivo* perfusion model, PBMP model	12	P-m, P-t, and P-f	AB, VB, AF, brain, gut, kidney, liver, and RoB	MP, UCP	Reduce dose, increased frequency	Van Der Heijden et al. (2024)
Opioids									
Fentanyl	iv. or epid	MATLAB/Simulink	Caco-2 assay	21	P-m, P-t, and P-f	AB, VB, brain, gut, kidney, liver, and RoB	MP, UCP	NA	Shum et al. (2021)
Methadone	po	Simcyp	*Ex vivo* perfusion model	35	P-m, P-t, P-f	Full fetal PBPK model	MP, UCP	Dose adjustment not available	Badhan and Gittins (2021)
THC	iv. or inh	Simcyp	Physicochemical properties	12	P-m, P-t, P-f	AB, VB, AF, brain, gut, kidney, liver, and RoB	MP, UCP	Dose adjustment not available	Patilea-Vrana and Unadkat (2021)
Buprenorphine	sl	Simcyp V 21	Physicochemical properties	35	P-m, P-t, P-f	Full fetal PBPK model	MP, UCP	NA	van Hoogdalem et al. (2024)
Naltrexone	iv. or im	Pumas	Fick’s diffusion equation, Caco-2 assay	12	P-m, P-f	Fetal body	MP	Dose adjustment not available	Shenkoya et al. (2024)
Others									
Theophylline	iv. or po	MATLAB/Simcyp	Caco-2 assay, published data	12	P-m, P-t, and P-f	AB, VB, AF, brain, gut, kidney, liver, and RoB	MP, UCP	NA	Zhang et al. (2017), Zhang and Unadkat (2017)
Metformin	po	Simcyp V 20	Published data	35	P-m, P-t, and P-f	Full fetal PBPK model	MP, UCP, AF	NA	Abduljalil et al. (2022b)
Theophylline	iv. or po	Simcyp V 21	Published data	35	P-m, P-t, and P-f	Full fetal PBPK model	MP, UCP	NA	Abduljalil et al. (2022c)
Sildenafil	iv. or po	Simcyp V 21	*Ex vivo* perfusion model	35	P-m, P-t, and P-f	Full fetal PBPK model	MP, UCP	Dose adjustment not available	Macente et al. (2023)

Abbreviations: po., oral; iv., intravenous; im., intramuscular; inh., inhalation; epid., epidural; sl., sublingual; P-m, placenta (maternal); P-f, placenta (fetal); P-t, placenta (tissue); AB, arterial blood; VB, venous blood; MP, maternal plasma; UCP, umbilical cord plasma; AF, amniotic fluid; NP, neonatal plasma (around birth); QSPR, quantitative structure–property relationship model; PBMP, physiologically-based semi-mechanistic placenta; RoB, rest of body; THC: cannabinoid (−)-Δ9-tetrahydro-cannabinol; full fetal PBPK mode including adipose, bone, brain, gut, heart, kidney, liver, lungs, pancreas, muscle, skin, spleen, amniotic fluid, blood; BET, betamethasone; DEX, dexamethasone; NA, not applicable.

Although PBPK is a physiological PK simulation, it still relies on mathematical and mechanistic modeling. The goal of PBPK model development is to accurately reflect *the in vivo* physiological conditions. Studies have incorporated the fetal liver as an independent metabolic organ (Mian et al., 2020), considering the scaling of fetal drug-unbound fractions (Roelofsen et al., 2022) and adjusted parameters, such as fetal liver and kidney volumes and cytochrome P450 enzyme levels, to model fetal drug metabolism and clearance (Szeto et al., 2021). These efforts were aimed at improving the precision of fetal drug concentration predictions. Moreover, given the dynamic development of the placenta (Aplin et al., 2020) and the challenges in obtaining early pregnancy samples, future research should prioritize drug safety studies during early and mid-pregnancy to better guide maternal medication use. Further exploration of these approaches is crucial for future research as it will provide valuable evidence for clinical decision-making.

### 5.2 Computational approaches

Diverting from mechanistic approaches, novel mathematical techniques such as artificial intelligence and machine learning (ML) methods ranging from regression analysis to deep learning and neural networks have recently been utilized for the investigation of placental PK. One study used ML to stratify high-risk pregnant women based on biomarkers and electronic health data, providing insights into maternal vaccination and placental antibody transfer for personalized immunization (Wessel and Dolatshahi, 2024). Another study explored the placental transport of toxic substances using a uterine perfusion model and molecular docking (Zhang et al., 2024). Additionally, a quantitative structure–property relationship (QSPR) model optimized using Monte Carlo methods was developed for high-throughput screening of placental drug permeability (Vukomanović et al., 2024). Similarly, researchers have used eigenvalue analysis, a chiral-sensitive QSPR method combined with ML, to predict placental drug transfer, focusing on chirality and stereochemistry (Gomatam and Coutinho, 2024). These efforts highlight the growing role of computational approaches in advancing our understanding of placental PK and enhancing drug safety during pregnancy. Although the application of these techniques for the prediction of placental drug transfer is in its infancy, they are slowly gaining traction as powerful PK tools. However, it is apparent that novel mathematical techniques such as these have great potential to fill the knowledge gap in underrepresented populations and clinical scenarios, and future research initiatives should encompass these techniques.

## 6 Discussion

Placental pharmacokinetics refers to the study of how drugs are absorbed, distributed, metabolized, and excreted as they pass from the maternal to the fetal circulation via the placenta. As a selective barrier and active transport interface, the placenta plays a pivotal role in determining fetal drug exposure. Understanding placental pharmacokinetics is essential for optimizing medication use during pregnancy, ensuring maternal efficacy while minimizing fetal risk. To address the complexity of maternal–fetal drug transfer, a range of experimental and computational models have been developed to investigate the placental transport mechanisms and predict fetal exposure. Recent advances in *ex vivo* placental perfusion, organ-on-a-chip platforms, and *in silico* simulations offer promising avenues to improve the accuracy of fetal drug-exposure predictions.

### 6.1 Placental pharmacokinetic models: characteristics and applications

Current methods for placental pharmacokinetics include cell models, organ models, and *in silico*-guided models, each with its own distinct characteristics (Table 5). Monolayer cell models are convenient and rapid, but they oversimplify placental dynamics (Birch et al., 2018). *Ex vivo* and organ-on-chip systems offer greater physiological relevance, yet they face practical constraints (Pemathilaka et al., 2019a). *In silico* models, such as PBPK, allow quantitative predictions but rely on experimental data, highlighting the need for integrated approaches. A review of current studies shows that monolayer cell models are used to evaluate placental drug permeability and transporter-mediated transfer (Mian et al., 2020; Po et al., 2018), *ex vivo* and organ-on-a-chip systems quantify fetal drug exposure and pharmacokinetics (Freriksen et al., 2020; Zhang and Unadkat, 2017), and PBPK models support clinical dosing during pregnancy while predicting fetal drug exposure (Schneider et al., 2022; Abduljalil et al., 2022b).

**TABLE 5:** Summary of the characteristics of the various methods.

Methods	Advantages	Limitations	Applicable boundaries
Caco-2 cell model	Simple, cost-effective, and high-throughput screening	Lacks full placental physiology	Early drug permeability prediction
Trophoblast cell lines	Simple, cost-effective, and high-throughput screening	Monolayer cells differ from actual placental barrier	Study of placental transporter proteins
Placental cotyledon perfusion experiment	Retains full placental structure and function	Limited donors and demanding experimental conditions	Drug transport kinetics and metabolism studies
Organ-on-a-chip	Simulates dynamic placental environment, integrates multiple cell types	Technology lacks standardization, long-term stability issues	Placental drug transport, maternal–fetal barrier function, and nanomedicine evaluation
PBPK model	Integrates physiological parameters for PK	Relies on experimental data and needs clinical validation	Prediction of fetal drug-exposure during pregnancy

Based on the comparative evaluation of each method, model selection in practical research should be guided by the research objective, the required physiological relevance, and the available resources. Simplified *in vitro* systems, such as cell monolayers or co-cultures, are appropriate for high-throughput screening or preliminary mechanistic studies because of their reproducibility and efficiency. Studies that demand physiologically relevant transport dynamics benefit from *ex vivo* placental perfusion or organ-on-a-chip models, which preserve tissue architecture and flow conditions absent in conventional *in vitro* systems. Computational approaches, including PBPK modeling, can supplement experimental data by providing quantitative predictions across different scenarios and species. Careful alignment of model choice with study goals and experimental constraints enhances both the reliability and translational value.

### 6.2 Multi-model integration: strengthening predictive power and translational relevance

The integration of multiple models holds significant potential in enhancing predictive accuracy and individual risk assessment. For instance, multiple studies demonstrate that incorporating *ex vivo* placental perfusion parameters into PBPK modeling yields predictions within 2-fold of clinical observations, significantly improving fetal drug-exposure estimates compared to non-integrated models (Mian et al., 2020; Roelofsen et al., 2022; Schalkwijk et al., 2018; Van Der Heijden et al., 2024). Another study showed that incorporating BeWo cell transporter kinetics and placental transporter abundance (particularly efflux transporters) into PBPK models can improve the physiological relevance of fetal drug-exposure predictions, as evidenced by the maternal-to-fetal plasma ratio shifting from approximately 1.0 to 0.1 (Al-Majdoub et al., 2025). In addition, integrating omics data (transcriptomics and proteomics) with dynamic placenta-on-a-chip platforms could enhance our understanding of drug–nutrient interactions, transporter regulation, and placental metabolism. The synergy between these methods facilitates a more accurate representation of the complex physiological processes involved in drug transfer, metabolism, and response, ultimately paving the way for tailored, safer treatments, especially in sensitive populations such as pregnant women. Beyond studies focused solely on placental drug-transfer, modeling approaches developed in this domain may offer significant insights into fetal drug-exposure arising from maternal vaccination, prolonged pharmacotherapy, or environmental toxin exposure during pregnancy.

Interestingly, the integration of data-driven approaches with mechanistic PBPK models also represents a highly promising strategy in PK research, particularly for pregnant populations. ML and QSPR methods can estimate parameters that are difficult to obtain experimentally; these parameters can be incorporated into PBPK models to enhance physiological realism and predictive accuracy. For example, a hybrid deep learning-PBPK model has successfully predicted human PK profiles from chemical structures, generating reliable concentration–time curves (Li et al., 2024). AI-assisted PBPK models have also been applied in the delivery of nanomedicines, producing predictions that closely match experimental PK data (Chou et al., 2023). These studies collectively demonstrate that utilizing ML or QSPR to inform mechanistic models can strengthen model development, optimize predictive performance, and enhance translational relevance. The application of such integrative strategies to placental pharmacokinetics holds considerable promise for advancing the field.

These technologies can help clarify how drugs cross the placenta and guide safer treatment for pregnant women with conditions such as epilepsy, hypertension, or HIV, where treatment is often unavoidable. The valuable results obtained from these methods could not only support drug label modifications but also inform the development of evidence-based treatment guidelines. Placental perfusion studies integrated with PBPK modeling reveal that doravirine exhibits a 55% reduction in maternal AUC during late pregnancy (40 weeks) (Bukkems et al., 2022), elevating treatment failure risk while predicting substantial fetal exposure. These findings have prompted multiple guideline bodies to re-evaluate its gestational use, shifting from “insufficient data, not recommended” toward “use with monitoring.” Similarly, quantified placental transfer kinetics of dexamethasone/betamethasone have enabled the precise prediction of maternal–fetal exposure dynamics (Van Der Heijden et al., 2024), advancing guidelines from fixed-dose to gestational age-adjusted regimens for optimal risk–benefit balance. In addition, a study combining Caco-2 cell models and placental perfusion experiments with PBPK modeling predicted that arterial umbilical concentrations of acetaminophen remain well below the recommended postnatal ductus arteriosus closure threshold (24.47 mg/L) (Mian et al., 2020). Regulatory agencies such as the Food and Drug Administration (FDA) and European Medicines Agency (EMA) increasingly accept PBPK modeling and may require placental transport assessment for drugs used in pregnancy in the future.

### 6.3 Future perspectives: gestational age and pathophysiology considerations

We have previously emphasized the need to investigate dynamic changes in the placenta during pregnancy, which are mainly driven by alterations in blood volume, blood flow velocity, tissue distribution, and the expression of metabolic enzymes and transporters. Nevertheless, few studies have specifically addressed this aspect, thus requiring greater attention in future research. Reviewing existing methodologies and models, several potential strategies may address this challenge, including (Be et al., 2025) conducting *ex vivo* placental perfusion using tissue samples from different gestational stages (early, mid, and late pregnancy) under ethically permissible conditions (Goeden and Bonnin, 2013); (Lupattelli et al., 2014) inducing pluripotent stem cells (iPSCs) to differentiate into trophoblasts, simulating developmental states across pregnancy stages (early trophoblasts vs. mature STBs) (Horii et al., 2016); (Subramanian et al., 2023) placenta-on-a-chip platforms, simulating the endocrine environment of different gestational stages by regulating key hormone levels (progesterone, estrogen, and hCG) in culture media (Mathiesen et al., 2025; Shojaei et al., 2021; Phillips et al., 2024). PBPK modeling can incorporate time-dependent parameters such as placental blood flow and transporter expression profiles to construct precise drug-use models for different gestational periods (Zhang et al., 2017) (Zhang and Unadkat, 2017). Furthermore, integrating *in vitro* data from different gestational stages with PBPK simulations offers a promising strategy to account for pregnancy-related variability.

In addition to gestational age-related dynamic changes, various pathological conditions can also markedly alter the placental structure and function, thereby influencing drug transfer. Preeclampsia [reduced placental perfusion and abnormal spiral artery remodeling (Roberts and Escudero, 2012; Yagel et al., 2022; Harmon et al., 2016)], gestational diabetes mellitus [hyperglycemia-induced upregulation of inflammatory mediators and altered transporter expression (Calvo et al., 2024)], and intrauterine infection [enhanced inflammatory response and increased tissue permeability (Meg et al., 2022)] are among the most common pregnancy complications associated with changes in villous stroma and vascular architecture. These pathological alterations can further modify the expression and activity of transporters and metabolic enzymes, ultimately affecting maternal–fetal drug transfer. To reproduce these pathological phenotypes *in vitro* or through modeling, several feasible approaches have been explored. For instance, *ex vivo* placental perfusion using tissues from pathological pregnancies [e.g., preeclampsia or GDM (Villota et al., 2021)] allows direct measurement of permeability differences under disease conditions. In cell-based and organ-on-a-chip models, preeclampsia, infection, or hyperglycemia can be mimicked by inducing hypoxia, adding pro-inflammatory cytokines (such as TNF-α or IL-6), or applying high-glucose culture conditions (Elzinga et al., 2023). Moreover, chip platforms can be adapted to modify shear stress, perfusion rates, or introduce pathological flow patterns to reflect abnormal spiral artery remodeling and reduced perfusion (Mosavati et al., 2020). Together, incorporating both pathophysiological states and gestational dynamics into placental models would greatly enhance their translational relevance for predicting drug disposition during pregnancy.

## 7 Conclusion

In this review, we describe the cell monolayer model, *ex vivo* placental perfusion experiments, placental-on-a-chip, PBPK model, and other *in vitro* research methods and discuss their advantages and disadvantages. The approach of a multi-model data fusion strategy (e.g., integrating a placenta perfusion experiment and PBPK modeling) is promising for the investigation of drug pharmacokinetics during pregnancy early in drug development, but further standardization and extension is needed. As technological fidelity improves and validation expands, placental drug modeling may become an essential, not optional, component of maternal–fetal pharmacokinetics.

## Glossary

AB: Arterial blood
ABC: ATP-binding cassette
ADME: Absorption, distribution, metabolism, and excretion
AF: Amniotic fluid
AECs: Amniotic epithelial cells
AMCs: Amniotic mesenchymal cells
BCRP: Breast cancer resistance protein (ABCG2)
BeWo: Human choriocarcinoma trophoblast cell line BeWo
BET: Betamethasone
CANA: Canagliflozin
CAI: Cellular accumulation index
CLI: Clearance index
CLp: Transplacental clearance
CTBs: Cytotrophoblasts
CTCs: Cytotrophoblast cells
CZP: Certolizumab pegol
DAPA: Dapagliflozin
DCs: Decidual cells
DEX: Dexamethasone
Dcot: Cotyledon clearance
EMA: European Medicines Agency
EMPA: Empagliflozin
ENT: Equilibrative nucleoside transporter
EVTBs: Extravillous trophoblasts
FDA: Food and Drug Administration
FTR: Fetal transfer rate
GS: GS-441524
HUVECs: Human umbilical vein endothelial cells
HPVECs: Human placental vascular endothelial cells
hTSCs: Human trophoblast stem cells
iNOS: Inducible nitric oxide synthase
IV: Intravenous
LMV: Lamivudine
LLOQ: Lower limit of quantification
LVFX: Levofloxacin
MBV: Maribavir
MCT: Monocarboxylate transporter
MP: Maternal plasma
MRPs: Multidrug resistance-associated proteins (ABCC family)
MVM: Microvillous membrane
NA: Not applicable
NP: Nanoparticle
OATs: Organic anion transporters
OATP2B1: Organic anion transporting polypeptide 2B1
OCTs: Organic cation transporters
OC: Oseltamivir carboxylate
OP: Oseltamivir phosphate
PBMP: Physiologically based mechanistic placenta
PBPK: Physiologically based pharmacokinetic
PEA: Peanut allergen
PDE1: Phosphodiesterase 1
PDE5: Phosphodiesterase 5
P-gp: P-glycoprotein (ABCB1)
PK: Pharmacokinetics
PlGF: Placental growth factor
po: Oral administration
P-m: Placenta (maternal)
P-f: placenta (fetal)
P-t: placenta (tissue)
QSPR: Quantitative structure–property relationship
Rmd: Remdesivir
RoB: Rest of body
SF: Surfactant
sEng: Soluble endoglin
sFlt-1: Soluble fms-like tyrosine kinase-1
SGLT2-i: Sodium-glucose cotransporter-2 inhibitor
sl: Sublingual
STB/STBs: Syncytiotrophoblasts
TEER: Transepithelial electrical resistance
THC: Cannabinoid (-)-Δ9-tetrahydro-cannabinol
TNF-α: Tumor necrosis factor alpha
TPTs: Transporter proteins
UCP: Umbilical cord plasma
VB: Venous blood
